# Triple diagnostics for early detection of ambivalent necrotizing fasciitis

**DOI:** 10.1186/s13017-016-0108-z

**Published:** 2016-10-11

**Authors:** Falco Hietbrink, Lonneke G. Bode, Louis Riddez, Luke P. H. Leenen, Marijke R. van Dijk

**Affiliations:** 1 Department of surgery, University Medical Center Utrecht, Utrecht, The Netherlands; 2 Department of Medical Microbiology, University Medical Center Utrecht, Utrecht, The Netherlands; 3 Department of Surgery, Karolinska Institutet, Solna, Sweden; 4 Department of Pathology, University Medical Center Utrecht, Utrecht, The Netherlands

**Keywords:** Necrotizing fasciitis, Early recognition, Triple diagnostics, Histology, Fresh frozen section

## Abstract

**Background:**

Necrotizing fasciitis is an uncommon, rapidly progressive and potential lethal condition. Over the last decade time to surgery decreased and outcome improved, most likely due to increased awareness and more timely referral. Early recognition is key to improve mortality and morbidity. However, early referral frequently makes it a challenge to recognize this heterogeneous disease in its initial stages. Signs and symptoms might be misleading or absent, while the most prominent skin marks might be in discrepancy with the position of the fascial necrosis. Gram staining and especially fresh frozen section histology might be a useful adjunct.

**Methods:**

Retrospective analysis of 3 year period. Non-transferred patients who presented with suspected necrotizing fasciitis are included. ASA classification was determined. Mortality was documented.

**Results:**

In total, 21 patients are included. Most patients suffered from severe comorbidities. In 11 patients, diagnoses was confirmed based on intra-operative macroscopic findings. Histology and/or microbiotic findings resulted in 6/10 remaining patients in a change in treatment strategy. In total, 17 patients proved to suffer necrotizing fasciitis. In the cohort series 2 patients died due to necrotizing fasciitis

**Conclusion:**

In the early phases of necrotizing fasciitis, clinical presentation can be ambivalent. In the present cohort, triple diagnostics consisting of an incisional biopsy with macroscopic, histologic and microbiotic findings was helpful in timely identification of necrotizing fasciitis.

## Background

Necrotizing fasciitis is a relatively rare disease, which describes a group of infections that comprises skin, soft tissue and muscle and swiftly can spread through fascial planes [1–3]. The disease can be rapidly progressive and can have devastating outcome with many patients not surviving the infection (up to 70 % mortality rate reported in past series) [2]. Early diagnosis followed by immediate and thorough surgical debridement of affected tissue is necessary to prevent mortality and curb the systemic effects from resultant sepsis. However, diagnosis in the early stages can be challenging [4, 5]. Patients with necrotizing fasciitis might be brought to the ICU because of their sepsis without known cause and later prove unresponsive to resuscitation therapy [6, 7]. In a systematic review, a close correlation between the percentage of initially missed cases and the mortality rate in the presented cohort series has been described [8]. A 75 % mortality reduction has been reported if operated within 12 h after onset [9–13]. Moreover, a mismatch between external signs and affected fascia has been mentioned. Thus, early recognition, timely surgery and thorough initial debridement are mandatory for survival [6, 8].

Over the last decade, mortality rate has decreased to 20-40 % in reported series [14–17]. Some have attributed this to the improved awareness for necrotizing fasciitis at general practitioners and ED-physicians, probably due to the attention that has been given to this disease in medical journals and general media [18]. Due to this improved awareness, patients are presented to the different surgical specialties in more early stages of their disease. This is a challenge for the treating surgeon, as local signs can be minimal and only become more prominent as the disease progresses [19]. In these early stages of necrotizing fasciitis, triple diagnostics is suggested to be a useful adjunct in obtaining a diagnosis [20]. We provide an algorithm that contributes in the early phases of these patients in which a fresh frozen section and Gram staining can be of paramount importance to the treating surgeon. Implementation of this algorithm was analysed.

## Methods

### Patients

A retrospective analysis was performed of all non-transferred patients, presented to the emergency department of the University Medical Centre Utrecht with suspected necrotizing fasciitis. Inclusion criteria were age >18 and incisional biopsy or operation performed under the suspicion of necrotizing fasciitis. No exclusion criteria were formulated. A waiver was granted by the Ethical Committee for retrospective data collection.

Comorbidity severity was scored according to the ASA (American Society for Anesthesiologists) classification. Patients are scored grade 1–4 in our hospital describing the pre-hospital situation, with the addendum that grade 5 and 6 (created for emergency surgery settings) are not used in our hospital as all critical ill patients will be in those categories upon presentation. Mortality was recorded.

Deep tissue pain, hypoesthesia, purple skin changes, ecchymotic changes of intact skin indicate neural and vascular involvement and signify the need for immediate operative intervention without biopsy [19]. All other patients undergo incisional biopsy. Macroscopic findings that are suggestive for necrotizing fasciitis are summarized in Table 1. Findings that are suggestive for necrotizing fasciitis in fresh frozen sections or Gram stain are listed (Table 1). Only few studies describe their results on triple diagnostics, which makes a meta-analysis of this procedure not possible [20, 21]. To endorse the usefulness of triple diagnostics in necrotizing fasciitis, we questioned in what frequency its use had led to an altered treatment strategy in our hands.

**Table 1:** Characteristic findings suggestive for necrotizing fasciitis. Typical findings that can indicate necrotizing fasciitis during incision biopsy for macroscopic findings and findings on the fresh frozen section and Gram staining

Macroscopy	Fresh frozen section	Gram stain
• Dishwater pus • Lack of bleeding • Lack of tissue resistance • Grey necrotic tissue • Non-contracting muscles • Fascial oedema • Purple blisters on skin	• Necrosis of superficial fascia • Polymorphonuclear infiltration of the deep dermis and fascia • Fibrinous trombi of arteries and veins passing through the fascia • Angiitis with fibrinoid necrosis of vessel walls • Microorganisms within the destroyed fascia and dermis	• Microbes • With or without leukocytes • Group A Streptococci • Clostridium perfringens • Vibrio species

### Triple diagnostics: macroscopic findings

When necrotizing fasciitis is suspected, an incisional biopsy over the most suspected area is obtained via an longitudinal or incision in the Langer lines [6, 22, 23]. Classical signs indicative for necrotizing fasciitis are swollen tissue, dull grey necrotic tissue, grey fascia, lack of bleeding, small vessel thrombosis, “dishwater” pus, non-contracting muscle fibres and a positive “finger test” [24–26]. These macroscopic findings are pathognomonic and should prompt aggressive surgical debridement (Table 1). However, especially in the early phases of necrotizing fasciitis or immunocompromised patients, classical signs may not be present during biopsy at all or are present on a distant site from the external signs [27]. Merely oedema is no reason for thorough debridement. In these cases, triple diagnostics by biopsy might be an adjunct for both diagnosis and treatment. This was first coined in the early eighties for ambivalent cases of necrotizing fasciitis [19, 20]. Since then, it has been mentioned occasionally, but has not been given the place in diagnostics it deserves and even neglected in recent guidelines due to lack of large scale studies [26]. In ambivalent cases microbiological findings by urgent Gram staining and histopathological analysis by fresh frozen section of soft tissue should be obtained [6, 20, 22]. The sample should contain infected subcutaneous tissue, fascia and muscle of the affected area.

### Triple diagnostics: Gram staining

Fascia biopsy material is transported to the lab in a sterile container. For Gram staining, the tissue is fixed to a glass slide by alcohol or heating. For microscopy, x1,000 magnification (using an oil immersion objective lens (100×)) is used to assess the presence, Gram staining, characteristic arrangements and morphology of microorganisms.

Group A *Streptococci* (GAS, also called *Streptococcus pyogenes*) are Gram positive spherical cocci. In clinical specimens such as fascia biopsy material, they may appear as pairs or short chains. However, when they are grown in liquid media, they form the typical long chains. Polymicrobial infections are usually mixtures of aerobic and anaerobic bacteria, and therefore, many morphologically different microorganisms can be seen. Gram staining may even show more different microorganisms than are cultured eventually, as culturing anaerobic bacteria can be difficult due to specific growth requirements. Antibiotic therapy can be modulated according to the results of the Gram stain.

Gram staining and microscopy can be performed rapidly after arrival of the tissue in the lab, with a turn-around-time of approximately 30 min depending on the techniques used in the lab and the skills of the microscopist. Negative microscopy does not rule out the presence of microorganisms in tissue however; tissue should therefore be cultured as well. This also facilitates antimicrobial susceptibility testing on the causative microorganisms. Special care should be given to anaerobic microbes.

### Triple diagnostics: fresh frozen section

Especially in cases with only peri-fascial oedema and absence of macroscopic necrosis, a fresh frozen section is of the upmost importance. Fresh, non-fixed tissue from a true cut section including fascia is embedded in gel and frozen rapidly to about −20° C. With a cryostat sections of 6 to 9 micrometer are produced and stained with hematoxylin and eosin (H&E). This procedure takes 10 to 15 min. The most specific predictive finding is necrosis of the superficial fascia with fibrinous trombi of arteries and veins located in the fascia. The vessels walls can show signs of angiitis with fibrinoid necrosis of the walls. Both the fascia and the deep dermis often show infiltration of polymorphonuclear cells. If bacteria are present in large numbers, they can often be seen in the H&E staining [20, 28]. In macroscopically obvious cases of necrotic fascia, histology will only demonstrate non-specific necrosis and is not indicated. No data is available about under and over diagnosis using this method and microbiology and pathology findings should not replace clinical parameters. Nevertheless, the combination of the 3 modalities might provide the surgeon sufficient data to identify the correct patients as early as possible or to extent the exploration of the suspected area.

### Suggested approach

**Fig. 1:**
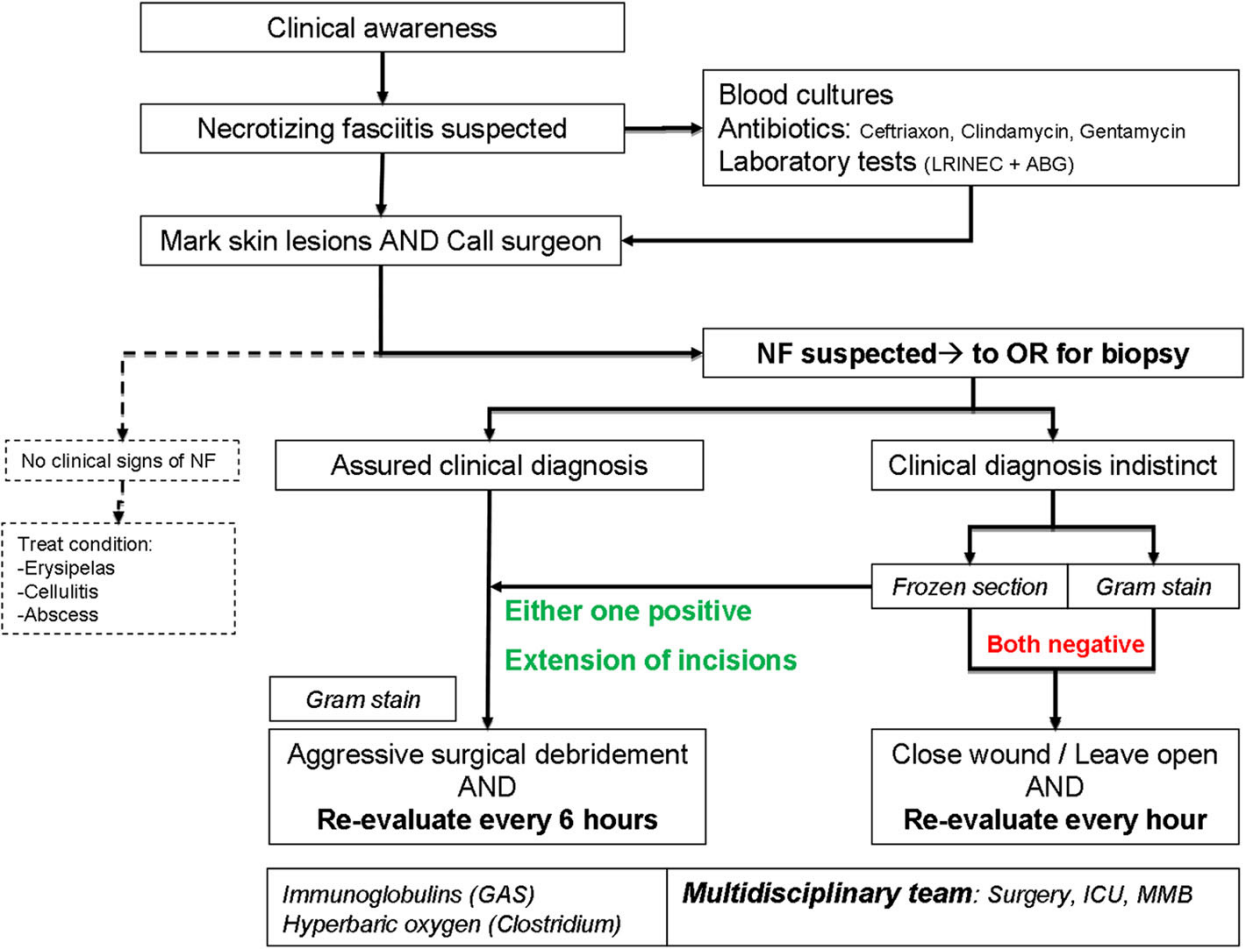
Clinical algorithm for suspected fasciitis. The algorithm used for gate specialties in patients with suspected necrotizing fasciitis. It consists of awareness, early surgical consultation and early initiation of treatment. When incision biopsy is indicated, the patient is transported to the operation room for further treatment. Treatment and aftercare is multidisciplinary

A treatment algorithm that might help in the management of these patients with ambivalent cases is postulated (Fig. 1). When there is a suspicion of necrotizing fasciitis, skin lesions are marked, blood cultures are drawn and laboratory tests are performed. Thereafter, broad spectrum antibiotics are initiated and should cover *Streptococcus* (Penicillin or 2nd/3rd generation Cephalosporin), Clindamycin (as toxin scavenger) and Gentamicin. The surgeon is consulted. Sepsis is treated immediately according to the Surviving Sepsis Campaign guidelines [29]. If clinical signs and symptoms in combination with laboratory tests are not suspicious, the patient is re-examined on set time points. However, if necrotizing fasciitis is suspected or cannot be ruled out, the patients consent is obtained for all possible scenario’s (debridement, amputation, intensive care and ventilator support and dialysis) and the patient is brought to the operation room for biopsy as soon as possible [30].

An incision is made over the most prominent skin marks or spot that is most painful. If during this procedure the diagnosis can be confirmed macroscopically, this prompts aggressive debridement. An incision biopsy for Gram stain is obtained, but should not delay further surgical control of the tissue when there are macroscopic findings of necrosis. Macroscopic necrosis frequently hampers the interpretation of histology and fresh frozen section is considered less useful in these cases.

However, if the diagnosis is indistinct by macroscopic findings (i.e. merely oedema), the biopsy is used for a Gram stain and fresh frozen section. If either one of them is suggestive for necrotizing fasciitis this prompts longitudinal extension of the incision. Skin marks can be misleading and necrotic lesions of the fascia can be found at distance after extension of the incision(s). When indicated by findings on histology of microbiology, aggressive debridement should follow of the entire affected area. Because either a positive or ambivalent macroscopic finding prompts further surgery, we prefer to perform the incision biopsy in the operation room.

After debridement, the wounds are left open, the patient is transported to the ICU for resuscitation and re-evaluated at set time points. When there are no indications for necrotizing fasciitis by macroscopy, Gram stain and fresh frozen section, the wound is either closed or left open when there is reasonable doubt. Re-evaluation takes place at set time points. Supportive therapeutic measures are initiated when indicated and based on mainly the Gram stain, such as immunoglobulins for GAS and hyperbaric oxygen in clostridium. Thereafter the patient is further treated by a multidisciplinary team, consisting of a surgeon, intensivist, microbiologist, physiotherapist, social worker, dietician and additional specialties depending on the location of the disease (i.e. plastic surgery, ophthalmologist, ENT-physician) [31]. When progression of necrosis is controlled, wounds are usually covered by vacuum devices until closure can be achieved.

## Results

**Fig. 2:**
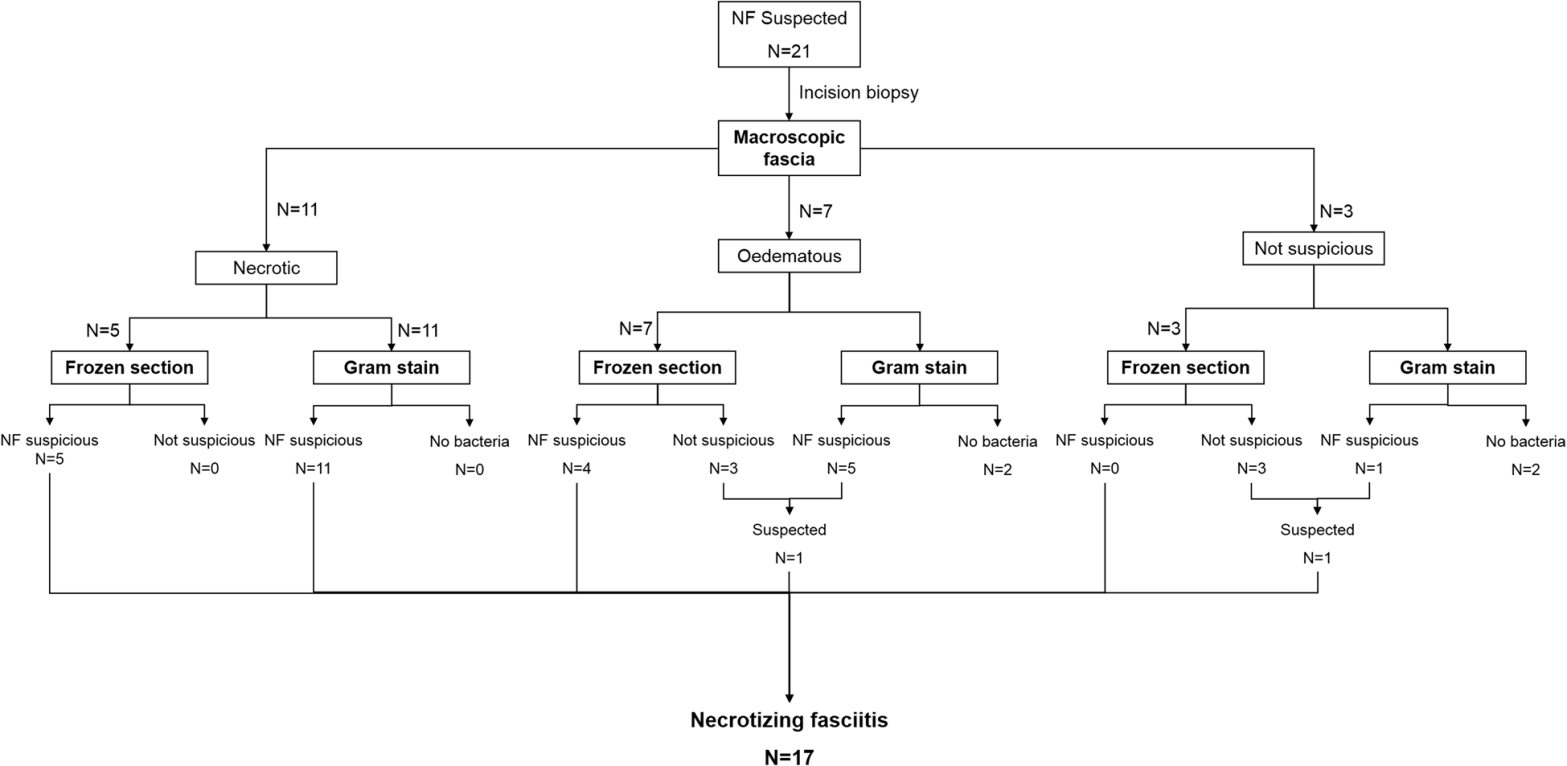
Patient flow. The flow of patients is depicted and decision making was based on macroscopic, histologic and microbiotic findings

In a three year period, 21 non-transferred cases were presented to the emergency department who underwent incision biopsy or operative debridement. Their average age was 53 (range 34–75) and most patients suffered from severe comorbidities (6 ASA I, 4 ASA II, 2 ASA III and 9 (47 %) ASA IV patients). In 11 patients, diagnoses was confirmed based on intra-operative macroscopic findings of fascial necrosis. There were 10 ambivalent cases with only macroscopic peri-fascial oedema or necrosis of the subcutaneous fatty tissue, in which fresh frozen section and Gram staining resulted in a change in treatment strategy in six patients. Based on macroscopic findings the surgeon would have ended the surgical procedure, but instead extended the incisional area and focal necrosis was found at a distant side in all 6 patients. Follow-up proved that 4/21 patients did not have necrotizing fasciitis (Fig. 2). Group A Streptococcus were found in 8 of the 17 patients with confirmed necrotizing fasciitis. Mortality due to necrotizing fasciitis was the outcome in two patients (12.5 %) and 2 additional patients died within the first 30 days after admission due to other pre-existing conditions (25 % total 30 day mortality).

## Discussion

In this cohort series we present the results of an algorithm which uses triple diagnostics for ambivalent cases of necrotizing fasciitis in the very early stages of the disease. In patients with an ambivalent presentation and no clear macroscopic necrosis of the fascia during incisional biopsy, the combination of a fresh frozen section and Gram staining altered treatment in 60 % of the cases. All of which later proved to be necrotizing fasciitis based on clinical follow-up. Mortality due to necrotizing fasciitis in this series was 12.5 % and overall mortality was 25 %, which is a fair result considering the large number of ASA IV classified patients.

The mortality rate in the present series is identical to the first report (12.5 %) on the use of histology in necrotizing fasciitis [20]. It was discussed if the relative low mortality was the result of the early operative debridement or could be attributed to the histology [19]. We feel that the use of histology and Gram staining results in more timely decision making and therefore early debridement and source control.

Necrotizing fasciitis is rare and heterogeneous in its presentation for body region as it can occur in every fascia and mimic many other infectious and non-infectious diseases. As a result, numbers per treating physician and expertise gained with this disease are often limited. In addition, physicians frequently find it a “scary” disease, because of its rapid progression and the necessary thorough debridement that might result in bad function and appearance [24].

Awareness is advocated in patients with sudden onset and rapid progression of a suspected infectious disease. Disproportional pain is often referred to as the common denominator in this disease and should trigger further investigation. More classical symptoms for necrotizing fasciitis, such as erythema, oedema, blisters, crepitus, and skin necrosis have been described in only 10–40 % of the cases and are seldom seen within the first 24 h [22, 32, 33]. The difficulty in recognition is further stretched by underlying conditions, for instance trauma, vascular disease, diabetic wounds or drug abuse [24]. Co-morbidities are found in nearly 50 % of patients with necrotizing fasciitis and frequently some form of trauma (blunt or penetrating) preceded symptoms. In the present series 55 % suffered severe co-morbidities such as congestive heart failure, renal insufficiency or acute leukemia. Although heterogeneous in its presentation, the philosophy of early identification and aggressive holistic treatment is uniform. This is often referred to as the “search and destroy” strategy [7, 21, 31, 34, 35]. In the presented cohort, a total of 4 patients were brought to the operation room based on clinical suspicion, who did not prove to have necrotizing fasciitis, This demonstrates the low threshold for incisional biopsy when necrotizing fasciitis is suspected, leading to a relative over treatment of patients with a less severe condition (i.e. erysipelas).

Proposed classifications are universal and either based on location or microbiology [36, 37]. Locations which are often affected are the trunk, extremities and the maxillofacial region. Frequently encountered specific locations include Fournier (perineum), Ludwig’s angina (submandibular) and Meleney’s synergistic gangrene (abdominal wall and/or post-operative) [24, 25, 38].

Classification by microbiology covers all locations, although some locations are more associated with a specific type than others. Type 1 accounts for 55–90 % of all cases and consists of a polymicrobial flora [24, 26, 38, 39]. Fournier is often associated with type 1 necrotizing fasciitis. Type 2 consists of a mono-microbial flora, of which necrotizing fasciitis with Group A Haemolytic Streptococcus (GAS, also called *Streptococcus pyogenes*) is the most important one. Other suggested classification groups are type 3 for virulent Gram negative bacilli (i.e. *Vibrio species*) and type 4 for fungi and yeasts (i.e. *Cryptococcus* or *Candida species*) [40]. Microbiological findings, and thus classification types, are highly geographically dependant. For instance, *Vibrio species* is mostly situated in Asia, while methicillin-resistant *Staphylococcus aureus* (MRSA) is seldom seen in the northern region of Europe [41, 42]. In order to combine multiple aetiologies, it has been proposed to integrate all types of necrotizing fasciitis like entities in the diagnose of severe necrotizing soft tissue disease as therapy is similar [43]. In addition, the potential whole body presentation causes many different medical specialties to be confronted with necrotizing fasciitis, resulting in more scattered experience. This stretches the need for a universal treatment algorithm [34, 44, 45].

To aid in the identification of patients with necrotizing fasciitis, several adjuncts have been described. A large base deficit or high Laboratory Risk Indicator for Necrotizing Fasciitis (LRINEC) score have been suggested to increase the possibility of a patient having necrotizing fasciitis, however, they are not tools to provide a definitive diagnosis [16, 32, 33, 46, 47]. Their values may provide insight in the severity of disease, however, sensitivity remains low [7, 48, 49].

Imaging studies might provide additional information. Although air in the fascial planes is seldom present in the early stages and fascial fluid collections are not always seen. Moreover, CT-scanning might provide information about underlying conditions in cases for necrotizing fasciitis in the maxillo-facial area or trunk (Fournier), such as diverticulitis or abscesses. Some clinics have incorporated CT-scanning in their standard work-up for hemodynamic stable patients with fasciitis to screen for underlying pathology. In certain cases CT helps to evaluate the extent of tissue infection showing swelling, inflammation and gas formation.

MRI scanning proves to have the highest sensitivity and specificity [50]. However, MRI scanning may not be desirable in all patients or available in all hospitals. Furthermore, the exact contribution of imaging modalities in the early stages of necrotizing fasciitis is still under debate and should always be correlated with the clinical presentation [48, 51, 52]. In more advanced cases treatment should not be delayed for imaging. Taken together, clinical suspicion should outweigh both laboratory and imaging adjuncts for the diagnosis of necrotizing fasciitis, especially in the early stages of the disease, where the therapeutic yield of debridement is the greatest [9]. Clinical suspicion can be supported by fresh frozen section and Gram staining during incisional biopsy and might result in more timely identification of this life threatening condition.

## Conclusion

With improved awareness, a challenge arises with the early and correct identification of necrotizing fasciitis. Signs and symptoms might be absent or misleading, as prominent skin marks might not be the place of fascial necrosis. This emphasizes the importance of adequate algorithms and treatment protocols for *all* medical specialties that might encounter necrotizing fasciitis. Identification and debridement as soon as possible and aggressive enough are the major contributors for survival. Therefore, triple diagnostics which include a fresh frozen section and Gram staining might be an important adjunct in early ambivalent stages of suspected necrotizing fasciitis.
